# Larvicidal Potential of Caribbean Plants

**DOI:** 10.1155/2023/5518863

**Published:** 2023-08-26

**Authors:** Rhaheem N. A. Layne-Yarde, Simone L. Sandiford

**Affiliations:** ^1^Department of Basic Medical Sciences, Pharmacology and Pharmacy Section, The University of the West Indies Mona, Kingston 7, Jamaica; ^2^Mosquito Control and Research Unit, The University of the West Indies, Mona, Jamaica

## Abstract

Mosquitoes are vectors for numerous arboviruses such as dengue, chikungunya, and Zika which continue to negatively impact the health of Caribbean populations. Within the region, synthetic insecticides are primarily used to control mosquito populations. In many countries however, these compounds are becoming less effective due to resistance, and they may also be harmful to the environment. Thus, there is a significant need for the development of alternative agents to combat the mosquito threat in the Caribbean. Worldwide, botanical-based products are being increasingly investigated for vector control because they are environmentally friendly and are often highly effective mosquitocidal agents. Although the botanical diversity within the Caribbean is remarkable, work on plant biopesticides in the region remains limited. The aim of this review, therefore, is to discuss the use of Caribbean botanical extracts as larvicidal agents. Additionally, we highlight the need for future work in this area which may subsequently lead to the implementation of transformative public health policies.

## 1. Introduction

The Caribbean is rich in botanical biodiversity with over 10,000 individual species having been discovered to date [1]. Located between North and South America, it consists of three island groups: The Bahamas, the Greater Antilles, and the Lesser Antilles. The Greater Antilles is comprised of the islands found in the western regions of the Caribbean Sea, whereas the Lesser Antilles is a group of smaller islands forming an archipelago in the eastern Caribbean Sea. The region has many endemic plants with a rich history of ethnomedical uses. Interestingly, many of these understudied species have phytochemical compositions which may be of great interest to researchers in the field of tropical medicine.

Although part of the same region, the islands of the Caribbean vary by geological evolution, and as such, their flora and fauna differ significantly. Not surprisingly, endemic plants are most abundant on the larger and more diverse continental islands of the Greater Antilles such as Cuba and Hispaniola [2, 3]. As the phytochemical profile of plants is influenced by factors such as location as well as environmental stress [4], variations may also be seen throughout the islands. The most abundant components of the leaf essential oils of *Canella winterana* (L.) Gaertn (Canellaceae) from the Dominican Republic, for instance, were myrcene, caryophyllene, and cis-ocimene [5], whereas those from the same plant in Guadeloupe were myrcene, (E)-*β*-farnesene and 2,3-methylenedioxy anisole safrole [6]. Likewise, analysis of the essential oils from Jamaican *Hyptis verticillata* Jacq. (Lamiaceae) revealed that the compound aromadendr-1(10)-en-9-one (30.7%) was the major constituent [7], while a similar study reported that cadin-4,10(15)-dien-3-one (14.8%) and isocaryophyllene epoxide (14.4%) were the most abundant compounds in the Cuban variety [8]. Selective ecological pressures over centuries have led plants to produce secondary metabolites to combat and defend against herbivorous insects [9]; therefore, it is not surprising that increased toxic activity is sometimes observed when local plant extracts are used as insecticides against some local insect species [10].

In the region, the productivity of many Caribbean territories has been severely impacted by epidemics of mosquito-borne diseases caused by arboviruses primarily transmitted by *Aedes aegypti* (Linnaeus) mosquitoes. These include cyclic outbreaks of dengue, and in more recent years, chikungunya and Zika. The unfortunate lack of effective vaccines or antiviral agents means that control of the mosquito vector is of paramount importance. To compound matters, the existing synthetic insecticides used for mosquito vector control are becoming less effective coupled with the fact that they may also cause harm to the environment. In Jamaica, *Ae. aegypti* mosquitoes have been found to be resistant to common insecticides such as permethrin and malathion [11], and mutations in voltage-gated sodium channels are thought to be a cause for pyrethroid resistance [12]. Additionally, resistance in *Ae. aegypti* mosquitoes to temephos was observed in Cuba [13] and in the Lesser Antilles where they were reported to be highly resistant to dichlorodiphenyltrichloroethane (DDT) [14]. Due to increasing worldwide use, there is now compelling evidence that botanical-based substances possess potent mosquitocidal properties [15]. Yet, work in this area in the region remains limited as these agents continue to be used primarily as mosquito repellents and not for their mosquitocidal activity. The aim of this review is therefore to discuss studies that have evaluated larvicidal activity of plants from the Caribbean as well as to identify research gaps that currently exist in the region.

## 2. Search Strategy

The scientific databases of PubMed (https://pubmed.ncbi.nlm.nih.gov/ (accessed on 25 February 2023)) and Google Scholar (https://scholar.google.com/ (accessed on 25 February 2023)) were searched for peer-reviewed literature relevant to this topic. A list of keywords and phrases used to initiate the searches included “larvicidal essential oils,” “Caribbean essential oils as larvicides,” and “larvicidal potential of essential oils”. Articles that involved larvicidal activity of Caribbean plants were included. The review focused on English-language papers but also included literature published in Spanish.

## 3. Larvicidal Activity of Plants from the Greater Antilles

Only one island of the Greater Antilles, Cuba, has reported larvicidal activity of botanical agents. Proactive public health policies have limited the mosquito threat in that country, and as such significant interest has been placed on the development of alternatives to traditional pesticides [16]. Being the largest island in the Caribbean, Cuba is also home to a vast array of unique endemic plant species, and studies have begun to assess their larvicidal potential. The essential oils from the leaves of *Eugenia melanadenia* Krug & Urb*. var. melanadenia* (Myrtaceae) and *Psidium rotundatum* Griseb (Myrtaceae) both Cuban endemic plants, were investigated against an *Ae. aegypti* reference laboratory strain from the Caribbean epidemiology centre for larvicidal activity [17]. The LC_50_ and LC_95_ values for *E. melanadenia* were 0.0085% and 0.0104%, while that of *P. rotundatum* were 0.0063% and 0.0071% [17] (Table 1). According to Komalamisra et al., essential oils with LC_50_ values between 50 and 100 ppm are considered effective, while those with LC_50_ values under 50 ppm are highly effective [18]. As such, both oils can be classified as effective larvicidal agents. Although phytochemical analysis was not done in this study, other researchers have demonstrated that the major chemical constituents of *E. melanadenia* were 1,8-cineole (45.3%), terpinen-4-ol (10.6%), p-cymene (8.2%), P-eudesmol (7.0%), and a-terpineol (6.7%) [19]. Interestingly, work performed in Egypt showed that 1,8-cineole was an ineffective larvicide against another mosquito species, *Culex pipiens* (Linnaeus) [20]. Other studies have also suggested that 1,8-cineole has low larvicidal activity against *Aedes aegypti* [21]. In contrast, terpinen-4-ol was shown to have significant larvicidal activity against both *Anopheles* and *Culex* species [22] in India. Similarly, chemical analysis of *P. rotundatum* revealed that *α*-pinene (18.3%) and 1,8-cineole (28.0%) were its major components [23]. In studies conducted in Lebanon, *α*-pinene was shown to possess potent larvicidal activity against the *Cx. pipiens* [24]. Therefore, the larvicidal activity demonstrated by both plants could be a result of synergistic effects of the chemical constituents.

Leyva et al. assessed the effect of the essential oils from *Piper auritum* H.B.K (Piperaceae), *Pimenta racemosa* (Mill.) J. Moore (Myrtaceae), *Chenopodium ambrosioides* L. (Amaranthaceae), and *Piper aduncum* L. (Piperaceae) against the established *Ae. aegypti* Rockefeller laboratory strain [25]. *Piper auritum*, *P. racemosa*, and *C. ambrosioides* were highly effective larvicides with LC_50_ values below 50 ppm, while *P. aduncum* can be classified as effective [25] (Table 1). In the same study, safrol (93.2%) was identified as the major component of the essential oils from *P. auritum*, the most effective oil assayed in the study. Intriguingly, safrol showed the greatest larvicidal activity against laboratory strains of both *Ae. aegypti* and *Cx. pipiens* in work conducted in the Republic of Korea [26]. Thus, these studies suggest that safrol is the active larvicidal agent from the essential oil of *P. auritum*.

The essential oil from *Pinus tropicalis* Morelet (Pinaceae) referred to as turpentine oil showed significant developmental inhibition and larvicidal death in *Ae. aegypti* mosquitoes [27]. The turpentine oil was reported to have a greater inhibitory effect on the *Ae. aegypti* Rockefeller strain when compared to the “San Miguel del Padrón 2011” *Ae. aegypti* field strain from Cuba. The LC_50_ and LC_95_ values of the modified turpentine oil tested against the Rockefeller strain were 0.0023 mg/mL and 0.0043 mg/mL, respectively, while the LC_50_ and LC_95_ values against the “San Miguel del Padrón 2011” field strain were 0.0058 mg/mL and 0.011 mg/mL [27] (Table 1). These results indicate that turpentine oil is a highly effective larvicidal agent based on the classification of Komalamisra et al. [18] and warrant further investigation of the constituents of the oil. Although chemical analysis was not performed in the study, reports from Greece suggest that essential oils from plants of the Pinus genus predominantly contain the monoterpene compounds *α*-pinene, *β*-pinene, and limonene as well as the sesquiterpenoid compounds germacrene D or *β*-caryophyllene [28].

Another study from Cuba involved the investigation of the larvicidal potential of *Melaleuca quinquenervia* (Cav.) S.T. Blake (Myrtaceae) against field *Ae. aegypti*, *Aedes albopictus* (Skuse) and *Culex quinquefasciatus* (Say). Importantly, *M. quinquenervia* essential oil was shown to be a highly effective larvicidal agent against field mosquitoes and was most prominent against *Cx. quinquefasciatus* with relatively low values of 0.0021% and 0.0064% [29]. LC_50_ and LC_90_ values obtained against *Ae. aegypti* were 0.0047% and 0.014%, respectively, and *Ae. albopictus* 0.0049% and 0.0089% respectively [29] (Table 1). This highlights the fact that some essential oils may be more effective against some mosquito species when compared to others. The major chemical components of *M. quinquenervia* were shown to be 1,8-cineole, *α*-pinene, *β*-pinene, aterpineol, limonene, and the hydroxylated sesquiterpenoid viridiflorol.

Work conducted on the essential oils of *Eucalyptus globulus* Labill (Myrtaceae) and *Bursera graveolens* (Kunth) Triana & Planch (Burseraceae) demonstrated their larvicidal activity against the *Ae. aegypti* Rockefeller strain and field strains from multiple species. Interestingly, the larvicidal activity of *E. globulus* was greater against the *Ae. aegypti* Cuban field strain when compared to the *Ae. aegypti* Rockefeller laboratory strain [10] (Table 1), indicating that in some instances, local plants may have greater activity against local mosquitoes. When comparing the activity of the oils against the various field strains, *E. globulus* was more effective against *Ae. aegypti* and *Cx. quinquefasciatus* field strain and *B. graveolens* against *Ae. albopictus* [10] (Table 1). These results support the fact that the activity of the oils varies with mosquito species. The major constituents of the essential oils from *E. globulus* were p-cymene, *γ*-terpinene and 1,8-cineol, while limonene and *β*-elemene were the major components in the essential oils from *B. graveolens* [10].

In a similar study, the essential oils from *Piper aduncum* subsp. *Ossanum* (Piperaceae) and *Ocimim basilicum* L. (Lamiaceae) were tested for their larvicidal activity against the *Ae. aegypti* Rockefeller strain as well as field strains of *Ae. aegypti*, *Ae. albopictus* and *Cx. quinquefasciatus* [30]. The essential oils from *P. aduncum* subsp*. Ossanum* were most effective against the Rockefeller strain when compared to the other strains. It was previously reported from other studies in Cuba that camphor and camphene were the major components of the essential oils from this plant [31]. On the other hand, *O. basilicum* essential oils were most effective against the *Ae. albopictus* field strain (Table 1). Phytochemical analysis was not performed; however, reports from studies conducted in India found that methyl chavicol and methyl eugenol were the most abundant constituents in the essential oils of *O. basilicum* [32]. Further work needs to be done to determine if the Cuban variety of *O. basilicum* also contains similar constituents.

Phytochemical studies on the essential oils from *Croton linearis* Jacq. (*Euphorbiaceae*) revealed the presence of 1,8-cineole (26.66%), sabinene (9.37%), and 10-epi-*γ*-eudesmol (6.83%) as its major constituents [33]. The larvicidal activity of the whole essential oil from *C. linearis* was compared to a nanoemulsion preparation of the same oil. The LC_50_ and LC_90_ values for the whole essential oil were found to be 64.24 ± 0.31 *μ*g/mL and 143.85 ± 5.23 *μ*g/mL, respectively. The nanoemulsion preparation was more potent than the whole essential oil with a LC_50_ value of 17.86 ± 0.11 *μ*g/mL and a LC_90_ value of 62.86 ± 2.18 *μ*g/mL (Table 1) because of increased water solubility resulting in greater bioavailability [33].

## 4. Larvicidal Activity of Plants from the Lesser Antilles

Only two islands of the Lesser Antilles have reported studies on the larvicidal activity of plants. In Martinique, *Alpinia zerumbet* (Pers.) B.L. Burtt & R.M.Sm. (Zingiberaceae) was investigated for its toxic activity against the *Ae. aegypti* Société Béninoise d'Electricité strain [34]. Reports from India have indicated that plant species from the Zingiberaceae family are effective insecticides against moths [35]. Of all the plants of the Alpina genus, this plant is the most common in Martinique, and the phytochemical composition of its essential oils is known to vary depending on the geographical location. Some of the key constituents from the Martinique variety include terpinen-4-ol (14.7 to 22.6%), p-cymene (2.1 to 8.8%), *γ*-terpinene (6.9 to 14.7%), sabinene (7.0 to 16.5%), eucalyptol (17.4 to 25.2%), and *α*-terpinene (1.9 to 8.1%) [34]. For the Cuban variety, Mendiola et al. found that the major chemical components were the sesquiterpene compound viridiflorol (32.2%), terpinen-4-ol (14.1%), caryophyllene oxide (6.9%), and eucalyptol (5.0%) [36]. Essential oils from the flowers of the plant showed larvicidal activity at a concentration of 1% (Table 1) against the *Ae. aegypti* mosquito [34], indicating that it is not a very effective larvicidal agent. This lack of activity could be a result of several factors such as the strain that was tested and the fact that the chemical composition of the essential oil varies between different plant parts [37]. It should be noted, however, that in a Brazilian study in which the larvicidal activity of the leaf essential oils of *A. zerumbet* were tested against an *Ae. aegypti* laboratory strain from Ceará, Brazil, an LC_50_ value of 313 ppm was reported. Both studies, therefore, suggest that *A. zerumbet* essential oil is not very effective when compared to other leaf oil extracts [38].

In Trinidad, Chariandy et al. investigated the larvicidal activity of various plants using different solvents to obtain the plant extracts [39]. Using multiple solvents for extraction is beneficial as more compounds with varying polarities can be obtained from the plant. These differing polarities are due to key structural differences between compounds which may impact their biological effects. The petroleum ether and ethyl acetate plant extracts are from *Justicia pectoralis* Jacq. (Acanthaceae) were reported to be the most effective larvicidal agents when compared to *Manihot utilissima* Pohl (Euphorbiaceae) and *Stachytarpheta jamaicensis* Vahl (Verbenaceae). However, neither LC_50_ values nor chemical constituents were reported. The three plants were also reported to possess growth-retardant properties which would inhibit the mosquito larvae from progressing further in the mosquito life cycle. Other phytochemical studies on *J. pectoralis* conducted in Brazil revealed the presence of coumarins, alkaloids, and triterpenoids [40] which are secondary metabolites produced by the plant and may have been responsible for the mosquitocidal activity observed.

In another study, conducted in Trinidad, the larvicidal activity of methanol, acetone, and aqueous leaf extracts of *Cordia curassavica* (Jacq.) Roem & Schult (Boraginaceae), *Azadirachta indica* A. Juss (Meliaceae), *Mangifera indica* L. (Anacardiaceae), *Rhizophora mangle* L. (Rhizophoraceae), *Avicennia germinans* (L.) L. (Acanthaceae), and *Languncularia racemosa* (L.) C.F. Gaertn (Combretaceae) were evaluated [41]. Of the tested plants, the extracts of *C. curassavica* had the lowest LC_50_ of 758 mg/L for the methanol extract, and the highest was from *M. indica* with an LC_50_ value of greater than 20,000 mg/L against the *Ae. aegypti* larvae (Table 1). These values suggest that none of the tested extracts were effective larvicidal agents.

## 5. Significance of Studies

Although work from only three Caribbean countries has been reported in this review, it is evident that the use of botanicals as larvicidal agents is an area that is slowly gaining momentum. Cuba is at the forefront in the region, and multiple studies evaluating the larvicidal activity of essential oils have been conducted. Based on the LC_50_ values listed in Table 1, results obtained with essential oils are superior to that of other plant extracts similar to what has been reported elsewhere [42]. Additionally, as seen with studies targeting multiple species of field mosquitoes, the toxic effects of essential oils vary significantly with different mosquito species. Further emphasis should be placed on investigating combinations of different oils if targeting multiple species in one area is necessary.

## 6. Present Applications of Plant Extracts against Mosquitoes

Over the past hundred years, plant oils have been applied to the skin to repel insects. This practice is commonplace in the Caribbean especially during outbreaks of arboviral diseases. Applying botanical oils to the skin is a simple yet effective way to deter mosquitoes from biting and would therefore reduce the incidence of disease spread and contraction. Other ways people have been taking advantage of plant aromas are by placing botanical incense around the household. In the Caribbean, some incenses are made of powder from the pyrethrum plants and have proven to be very effective repellents [43]. Nevertheless, the importance of mosquito repellents as a measure of mosquito control is often underscored.

## 7. Future Work

There is great botanical biodiversity amongst the islands, and as such, there are many currently undiscovered species with great potential as mosquitocidal agents. It would be worthwhile for researchers in the region to delve deeper and seek to potentially characterize the many plant species that show prominent toxic effects against the *Ae. aegypti* mosquito or any other mosquito species that may pose a threat. Although this review is focused on larvicidal activity, even fewer studies have examined the use of botanicals as adulticides [10, 29]. There is therefore a pressing need for the evaluation of botanicals as mosquitocidal agents to continue. Comparative studies between the crude essential oil extracts and individual isolated compounds from the oils would give further insight into the specific phytochemicals responsible for the observed activity. Since there have been relatively few Caribbean countries involved in these studies, there are still likely many discoveries left to be uncovered.

In addition, determining the mechanism by which the oils result in toxicity would be a crucial and necessary step to fully weaponize the effects of the oils against mosquitoes. Essential oils are thought to work via multiple mechanisms including modification of GABA receptors and binding to octopamine receptors [44]. Current synthetic insecticidal agents work via many different mechanisms including acetylcholinesterase inhibition (organophosphates) [45] and activity at sodium-gated channels (pyrethroids) [46]. There is still much left to learn about how mosquitoes acquire resistance to these mechanisms and how to combat this issue. Hence, knowing the mechanisms of action of the oils could further increase the potency of combined formulations of multiple oils or oils blended with synthetic insecticides where the toxic activities would compound to produce synergistic effects. Chansang et al. elaborated on the significant adulticidal activity observed against *Ae. aegypti* mosquitoes when essential oils were combined with the insecticide pyrethrin [47]. These methods may become necessary in the future as insecticide resistance continues to increase in the Caribbean.

As essential oils are known to target mosquitoes in many different ways [48], future plans for mosquito biocontrol could encompass the spraying of plant extract formulations into and around open sources of water. Based on previously described behaviour of mosquitoes regarding where they are expected to lay eggs [49], this act could simultaneously accomplish up to three measures of mosquito biocontrol, namely, oviposition deterrence, direct ovicidal activity, and larvicidal activity. Due to the well-described toxic activities of these plant extracts against mosquitoes, people would be able to utilize low-concentrated formulations to great effect with little to no harm to themselves or the environment [15]. Researchers in the region would therefore need to investigate chemical formulations that would make this most feasible.

Lastly, companies in the United States have begun to market effective household insecticides whose active ingredients are phytochemicals extracted from plants. One such instance is the case with the house product Orange Guard^®^ which contains limonene. To fully benefit from the larvicidal potential of plant extracts, it is evident that more translational science needs to be done within the Caribbean. One way to achieve this is through multidisciplinary collaborations between researchers within the region. Additionally, the support of national, regional, and international stakeholders is of paramount importance as financial resources and manufacturing expertise are needed to bring these discoveries to market. Once achieved, the next stages in the region should focus on the development of effective botanical-based household agents that can be made available to the wider populations in the Caribbean.

## 8. Conclusion

Though limited in number, various studies have been carried out in many territories within the region. There is now much evidence to suggest that botanical-based agents can be used as alternatives to synthetic larvicidal products in the Caribbean. However, more impetus is still required before the use of these novel products can begin to be fully actualized and integrated into vector control programmes in Caribbean societies.

## Figures and Tables

**Table 1:** Chemical composition and larvicidal potency of botanical agents in the Caribbean.

Plant species	Plant family	Country	Plant extract	Major compound (s)	Targeted species	24 hr LC_50_ (ppm)	Reference
*Eugenia melanadenia*	Myrtaceae	Cuba	Leaf EO	ND	*Aedes aegypti* (CAREC)	85	[17]

*Psidium rotundatum*	Myrtaceae	Cuba	Leaf EO	ND	*Aedes aegypti* (CAREC)	63	[17]

*Piper auritum*	Piperaceae	Cuba	Leaf EO	Safrol	*Aedes aegypti* (ROCK)	17	[25]

*Pimenta racemosa*	Myrtaceae	Cuba	Leaf EO	4-terpineol, 1,8-cineol, eugenol, *α*-terpineol	*Aedes aegypti* (ROCK)	27	[25]

*Chenopodium ambrosioides*	Amaranthaceae	Cuba	Leaf EO	Carvacrol, *α*-terpineol, p-cymene	*Aedes aegypti* (ROCK)	35	[25]

*Piper aduncum*	Piperaceae	Cuba	Leaf EO	Dillapiole	*Aedes aegypti* (ROCK)	57	[25]

*Pinus tropicalis*	Pinaceae	Cuba	Turpentine oil	ND	*Aedes aegypti* (SDP)	5.8	[27]
					*Aedes aegypti* (ROCK)	2.3	

*Melaleuca quinquenervia*	Myrtaceae	Cuba	Leaf EO	1,8-cineol, *α*-pinene, *β*-pinene, aterpineol, limonene, viridiflorol	*Aedes aegypti* (FS)	47	[29]
					*Aedes albopictus* (FS)	49	
					*Culex quinquefasciatus* (FS)	21	
					*Aedes aegypti* (ROCK)	47	

*Piper aduncum* subsp. *Ossanum*	Piperaceae	Cuba	Leaf EO	ND	*Aedes aegypti* (FS)	36	[30]
					*Aedes albopictus* (FS)	57.3	
					*Culex quinquefasciatus* (FS)	59.5	
					*Aedes aegypti* (ROCK)	35.3	

*Ocimim basilicum*	Lamiaceae	Cuba	Leaf EO	ND	*Aedes aegypti* (FS)	47.5	[30]
					*Aedes albopictus* (FS)	9.5	
					*Culex quinquefasciatus* (FS)	31.4	
					*Aedes aegypti* (ROCK)	46.9	

*Eucalyptus globulus*	Myrtaceae	Cuba	Leaf EO	p-cymene, *γ*-terpinene, 1,8-cineol	*Aedes aegypti* (FS)	13.1	[10]
					*Aedes albopictus* (FS)	91.2	
					*Culex quinquefasciatus* (FS)	20.9	
					*Aedes aegypti* (ROCK)	27.6	

*Bursera graveolens*	Burseraceae	Cuba	Leaf EO	Limonene and *β*-elemene	*Aedes aegypti* (FS)	32.5	[10]
					*Aedes albopictus* (FS)	31.8	
					*Culex quinquefasciatus* (FS)	31.5	
					*Aedes aegypti* (ROCK)	10.1	

*Croton linearis*	Euphorbiaceae	Cuba	Leaf EO	1,8-cineole, sabinene, 10-epi-*γ*-eudesmol	*Aedes aegypti* (LS)	64.24 17.86^§^	[33]

*Alpinia zerumbet*	Zingiberaceae	Martinique	Flower EO	Terpinen-4-ol, p-cymene, *γ*-terpinene, sabinene, eucalyptol, *α*-terpinene	*Aedes aegypti* (SBE)	ND	[34]

*Justicia pectoralis*	Acanthaceae	Trinidad	Leaf ethyl acetate	ND	*Aedes aegypti* (LS)	ND	[39]

*Manihot utilissima*	Euphorbiaceae	Trinidad	Leaf ethyl acetate	ND	*Aedes aegypti* (LS)	ND	[39]

*Stachytarpheta jamaicensis*	Verbenaceae	Trinidad	Leaf ethyl acetate	ND	*Aedes aegypti* (LS)	ND	[39]

*Cordia curassavica*	Boraginaceae	Trinidad	Methanol Acetone Aqueous	ND	*Aedes aegypti* (FS)	758 >1,059 195,000	[41]

*Azadirachta indica*	Meliaceae	Trinidad	Methanol Acetone Aqueous	ND	*Aedes aegypti* (FS)	5,767 9,148.3 974,000	[41]

*Mangifera indica*	Anacardiaceae	Trinidad	Methanol Acetone Aqueous	ND	*Aedes aegypti* (FS)	>20,000 8,828 31,0000	[41]

*Rhizophora mangle*	Rhizophoraceae	Trinidad	Methanol Acetone Aqueous	ND	*Aedes aegypti* (FS)	>20,000 14,173 676,000	[41]

*Avicennia germinans*	Acanthaceae	Trinidad	Methanol Acetone Aqueous	ND	*Aedes aegypti* (FS)	10,465.5 >15,000 40,0000	[41]

*Languncularia racemosa*	Combretaceae	Trinidad	Methanol Acetone Aqueous	ND	*Aedes aegypti* (FS)	>20,000 >15,000 625,000	[41]

EO: essential oil; ND: not determined; CAREC: Caribbean epidemiology centre strain; ROCK: Rockefeller strain; SDP: San Miguel del Padrón 2011 strain; FS: field strain; LS: laboratory strain; SBE: Société Béninoise d'Electricité strain; ^§^nanoemulsion.

## References

[1] Maunder M., Leiva A., Santiago-Valentin E. (2008). Plant conservation in the Caribbean Island biodiversity hotspot. *The Botanical Review*.

[2] Caribbean Islands - Species (2019). CEPF. https://www.cepf.net/our-work/biodiversity-hotspots/caribbean-islands/species.

[3] Heileman S. Thematic Report for the Insular Caribbean Sub-Region. https://www.clmeproject.org/phaseone/dcenter/CLME%20Insular%20Caribbean%20Thematic%20Report%20draft%2016%20Feb%2007%5B1%5D.pdf.

[4] Prinsloo G., Nogemane N. (2018). The effects of season and water availability on chemical composition, secondary metabolites and biological activity in plants. *Phytochemistry Reviews*.

[5] Adams R. P., Zanoni T. A. (1990). Essential oils of plants from Hispaniola: 3. The leaf oil of Canella winterana(Canellaceae). *Journal of Essential Oil Research*.

[6] Abaul J., Udino L., Bourgeois P., Bessière J.-M. (1995). Composition of the essential oils of Canella winterana(L.) Gaertn. *Journal of Essential Oil Research*.

[7] Facey P., Porter R., Reese P., Williams A. D. (2005). Biological Activity and Chemical Composition of the Essential Oil from Jamaican *Hyptis verticillata Jacq*. *Journal of Agricultural and Food Chemistry*.

[8] Pino J., Bello A., Urquiola A., Marbot R. (2002). Essential oil of Hyptis verticillata Jacq. from Cuba. *Revista CENIC. Ciencias Químicas*.

[9] Trowbridge A. (2014). Evolutionary ecology of chemically mediated plant-insect interactions. *Ecology and the Environment*.

[10] Leyva M., Marquetti M., Montada D., Payroll J., Scull R. (2020). ACEITES ESENCIALES DE Eucalyptus globulus (labill) Y Bursera graveolens (kunth) Triana & planch PARA EL control DE MOSQUITOS DE IMPORTANCIA MÉDICA. *The Biologist*.

[11] Francis S., Saavedra-Rodriguez K., Perera R., Paine M., Black W. C., Delgoda R. (2017). Insecticide resistance to permethrin and malathion and associated mechanisms in Aedes aegypti mosquitoes from St. Andrew Jamaica. *PLoS One*.

[12] Francis S., Campbell T., McKenzie S. (2020). Screening of insecticide resistance in Aedes aegypti populations collected from parishes in eastern Jamaica. *PLoS Neglected Tropical Diseases*.

[13] Rodríguez M. M., Ruiz A., Piedra L. (2020). Multiple insecticide resistance in Aedes aegypti (Diptera: Culicidae) from Boyeros municipality, Cuba and associated mechanisms. *Acta Tropica*.

[14] Polson K. A., Rawlins S. C., Brogdon W. G., Chadee D. D. (2011). Characterisation of DDT and pyrethroid resistance in Trinidad and Tobago populations of Aedes aegypti. *Bulletin of Entomological Research*.

[15] Ghosh A., Chowdhury N., Chandra G. (2012). Plant extracts as potential mosquito larvicides. *Indian Journal of Medical Research.*.

[16] Alexander R., Anderson P. K. (1984). Pesticide use, alternatives and workers' health in Cuba. *International Journal of Health Services*.

[17] Aguilera L., Navarro A., Tacoronte Morales J. E., Leyva M., Marquetti M. (2003). Lethal effect of Cuban Myrtaceae on Aedes aegypti (Diptera Cuilicidae). *Revista Cubana de Medicina Tropical*.

[18] Komalamisra N., Trongtokit Y., Rongsriyam Y., Apiwathnasorn C. (2005). Screening for larvicidal activity in some Thai plants against four mosquito vector species. *The Southeast Asian Journal of Tropical Medicine and Public Health*.

[19] Pino J., Marbot R., Bello A., Urquiola A. (2003). Essential oil of Eugenia melanadenia Krug et Urb. From Cuba. *Journal of Essential Oil Research*.

[20] Zahran H. E.-D. M., Abdelgaleil S. A. M. (2011). Insecticidal and developmental inhibitory properties of monoterpenes on Culex pipiens L. (Diptera: Culicidae). *Journal of Asia-Pacific Entomology*.

[21] Silva W. J., Dória G. A., Maia R. T. (2008). Effects of essential oils on *Aedes aegypti* larvae: alternatives to environmentally safe insecticides. *Bioresource Technology*.

[22] Govindarajan M., Rajeswary M., Hoti S. L., Benelli G. (2016). Larvicidal potential of carvacrol and terpinen-4-ol from the essential oil of *Origanum vulgare* (Lamiaceae) against *Anopheles stephensi*, *Anopheles subpictus*, *Culex quinquefasciatus* and *Culex tritaeniorhynchus* (Diptera: Culicidae). *Research in Veterinary Science*.

[23] Pino J. A., Rosado A., Bello A., Urquiobi A., Garcia S. (1999). Essential oil of Psidium rotundatum Griseb. from Cuba. *Journal of Essential Oil Research*.

[24] Traboulsi A. F., Taoubi K., El‐Haj S., Bessiere J. M., Rammal S. (2002). Insecticidal properties of essential plant oils against the mosquito Culex pipiens molestus (Diptera: Culicidae). *Pest Management Science*.

[25] Leyva M., del Carmen Marquetti M., Tacoronte J. E. (2009). Actividad larvicida de aceites esenciales de plantas contra Aedes aegypti (L.)(Diptera: Culicidae). *Revista Biomédica*.

[26] Perumalsamy H., Kim N.-J., Ahn Y.-J. (2009). Larvicidal activity of compounds isolated from Asarum heterotropoides against Culex pipiens pallens, Aedes aegypti, and Ochlerotatus togoi (Diptera: Culicidae). *Journal of Medical Entomology*.

[27] Leyva M., del Carmen Marquetti M., French L., Montada D., Tiomno O., Tacoronte J. E. (2013). Efecto de un aceite de trementina obtenido de Pinus tropicalis Morelet sobre la biología de una cepa de Aedes (Stegomyia) aegypti Linnaeus, 1762 resistente a insecticidas. *Anales de Biologia*.

[28] Loannou E., Koutsaviti A., Tzakou O., Roussis V. (2014). The genus Pinus: a comparative study on the needle essential oil composition of 46 pine species. *Phytochemistry Reviews*.

[29] Leyva M., French-Pacheco L., Quintana F. (2016). *Melaleuca quinquenervia* (Cav.) S.T. Blake (Myrtales: Myrtaceae): natural alternative for mosquito control. *Journal of Tropical Medicine*.

[30] Leyva M., Marquetti M., Montada D. (2020). Actividad insecticida de los aceites esenciales de Piper aduncum subsp. ossanum y Ocimun basilicum sobre Aedes aegypti, Aedes albopictus y Culex quinquefasciatus. *Novitates Caribaea*.

[31] Abreu O., Pino J. (2008). Leaf oil composition of Piper aduncum subsp. ossanum(C. CD.) Saralegui from Cuba. *Natural Product Communications*.

[32] Joshi R. K. (2014). Chemical composition and antimicrobial activity of the essential oil of Ocimum basilicum L. (sweet basil) from Western Ghats of North West Karnataka, India. *Ancient Science of Life*.

[33] Amado J. R. R., Prada A. L., Diaz J. G., Souto R. N. P., Arranz J. C. E., de Souza T. P. (2020). Development, larvicide activity, and toxicity in nontarget species of the Croton linearis Jacq essential oil nanoemulsion. *Environmental Science and Pollution Research International*.

[34] Kerdudo A., Ellong E. N., Burger P. (2017). Chemical composition, antimicrobial and insecticidal activities of flowers essential oils of Alpinia zerumbet (Pers.) B.L.Burtt & R.M.Sm. from Martinique Island. *Chemistry & Biodiversity*.

[35] Singh K., Singh M., Sureja A., Bhardwaj R. (2012). Insecticidal activity of certain plants of zingiberaceae and araceae against Spodoptera litura F. and Plutella xylostella saunders in cabbage. *Indian Journal of Entomology*.

[36] Mendiola J., Pino J., Fernández-Calienes A., Mendoza D., Herrera P. (2015). Chemical composition and in vitro antiplasmodial activity of essential oils of leaves and flowers of alpinia zerumbet grown in Cuba. *Pharmacology*.

[37] Barra A. (2009). Factors affecting chemical variability of essential oils: a review of recent developments. *Natural Product Communications*.

[38] Cavalcanti E. S., Morais S. M., Lima M. A., Santana E. W. (2004). Larvicidal activity of essential oils from Brazilian plants against Aedes aegypti L. *Memórias do Instituto Oswaldo Cruz*.

[39] Chariandy C. M., Seaforth C. E., Phelps R. H., Pollard G. V., Khambay B. P. (1999). Screening of medicinal plants from Trinidad and Tobago for antimicrobial and insecticidal properties. *Journal of Ethnopharmacology*.

[40] Leal L., Silva A., Viana G. (2017). *Justicia pectoralis* , a coumarin medicinal plant have potential for the development of antiasthmatic drugs?. *Revista Brasileira de Farmacognosia*.

[41] Mohammed A., Chadee D. D. (2007). An evaluation of some Trinidadian plant extracts against larvae of Aedes aegypti mosquitoes. *Journal of the American Mosquito Control Association*.

[42] Manh H. D., Hue D. T., Hieu N. T. T., Tuyen D. T. T., Tuyet O. T. (2020). The mosquito Larvicidal activity of essential oils from Cymbopogon and eucalyptus species in Vietnam. *Insects*.

[43] Simaremare S. R. S., Hung C. C., Hsieh C. J., Yiin L. M. (2020). Relationship between organophosphate and pyrethroid insecticides in blood and their metabolites in urine: a pilot study. *International Journal of Environmental Research and Public Health*.

[44] Jankowska M., Rogalska J., Wyszkowska J., Stankiewicz M. (2018). Molecular targets for components of essential oils in the insect nervous system-a review. *Molecules*.

[45] Fukuto T. R. (1990). Mechanism of action of organophosphorus and carbamate insecticides. *Environmental Health Perspectives*.

[46] Narahashi T. (1969). Mode of action of DDT and allethrin on nerve: cellular and molecular mechanisms. *Residue Reviews*.

[47] Chansang A., Champakaew D., Junkum A. (2018). Synergy in the adulticidal efficacy of essential oils for the improvement of permethrin toxicity against Aedes aegypti L. (Diptera: Culicidae). *Parasites & Vectors*.

[48] Pushpanathan T., Jebanesan A., Govindarajan M. (2006). Larvicidal, ovicidal and repellent activities of Cymbopogan citratus Stapf (Graminae) essential oil against the filarial mosquito Culex quinquefasciatus (say) (Diptera: Culicidae). *Tropical Biomedicine*.

[49] Day J. F. (2016). Mosquito oviposition behavior and vector control. *Insects*.

